# The Inhibition of LPS-Induced Oxidative Stress and Inflammatory Responses Is Associated with the Protective Effect of (-)-Epigallocatechin-3-Gallate on Bovine Hepatocytes and Murine Liver

**DOI:** 10.3390/antiox11050914

**Published:** 2022-05-06

**Authors:** Tianle Xu, Run Liu, Hao Zhu, Yu Zhou, Tianxu Pei, Zhangping Yang

**Affiliations:** 1 Joint International Research Laboratory of Agriculture and Agri-Product Safety, Ministry of Education of China, Yangzhou University, Yangzhou 225009, China; tl-xu@yzu.edu.cn; 2 College of Animal Science and Technology, Yangzhou University, Yangzhou 225009, China; mx120200803@stu.yzu.edu.cn (R.L.); mx120210848@stu.yzu.edu.cn (H.Z.); 201902228@stu.yzu.edu.cn (Y.Z.); 3 College of Veterinary Medicine, Yangzhou University, Yangzhou 225009, China; 202001218@stu.yzu.edu.cn

**Keywords:** EGCG, bovine hepatocytes, hepatoprotective, MAPK/NF-κB signaling, antioxidant activity

## Abstract

This study aimed to evaluate whether (-)-epigallocatechin-3-gallate (EGCG) alleviates hepatic responses to lipopolysaccharide (LPS)-induced inflammation and oxidation. Isolated bovine hepatocytes and BALB/c mice were used for LPS challenge and EGCG pretreatment experiments in vitro and in vivo. LPS-challenged (6 μg/mL) hepatocytes exhibited increased levels of NF-κB (p65 and IκBα) and MAPK (*p*38, ERK, JNK) phosphorylation as well as increased binding activity of p65 to target pro-inflammatory gene promoters, and these effects were suppressed by pretreatment with 50 μM EGCG. Moreover, the reduction in Nrf2 signaling and antioxidant enzyme activities induced by LPS stimulation were reversed upon EGCG treatment. In vivo experiments demonstrated the protective role of EGCG in response to GalN/LPS-induced mortality and oxidative damage. Together, our results suggest that EGCG is hepatoprotective via inhibition of MAPK/NF-κB signaling and activation of the Nrf2 cascade. This information might help design strategies for counteracting hepatitis in ruminants and monogastric animals.

## 1. Introduction

Natural products have been proposed as promising strategies for the development of health-promoting agents [1,2,3]. As the main bioeffective components in green tea leaves, tea polyphenols (30% of dry weight) have received great attention from studies on their therapeutic effects against various pathological conditions [4,5]. Catechins, including epicatechin (EC), epicatechin-3-gallate (ECG), and epigallocatechin-3-gallate (EGCG), have been reported to mainly possess phenolic compounds in tea polyphenols [6]. Notably, the most proportioned EGCG among those catechins is closely related to health-boosting properties of tea polyphenols, such as antioxidation, anti-inflammation, and antidiabetic and cardioprotective properties, in vitro and in vivo [7,8]. Notwithstanding the fact that EGCG is controversial due to its negative effect on reproductive performance in swine and its degraded bioactivity by oral administration, EGCG is more likely to be beneficial to the health of humans and farm animals [9,10,11,12,13]. Therefore, more in-depth research is still required into the application and properties of EGCG.

Studies have demonstrated that increased circulation of lipopolysaccharides (LPS) leads to a systemic inflammatory response, including metabolic disorders of organisms and tissue damage in both monogastric and ruminant animals [14,15]. Among organisms, the liver is responsible for the clearance of LPS and contributes to cytokine production in the bloodstream [16]. For example, the endogenous LPS released in the case of subacute ruminal acidosis (SARA) has shown that overloaded LPS that passes by the liver elicits elevations in acute-phase proteins (APP) and immune biomarkers in ruminants [17]. Moreover, exogenous LPS, such as that released from bovine mastitis infected with Gram-negative pathogens that is translocated from circulation into the liver, is suggested to induce a storm of cytokine secretion (i.e., IL1β, TNFα, IL-6, and SAA3) [18]. The stimulation of multiple cell types by LPS shows that NF-κB and MAPK signaling are involved in the activation of inflammatory cascades [19,20,21]. During the defensive process that detoxifies LPS, the balance between free radicals and antioxidants is also disrupted and attributed to excessive production of reactive oxygen species (ROS) from the liver [22]. Failure to control the overabundance of ROS in the liver will result in oxidative stress, such as mitochondrial damage [23,24]. The prolonged duration of inflammatory or oxidative stress may result in hepatic failure and secreted cytokines or ROS by the inflammatory processes are suggested to influence animal production [25].

To uncover the preventive effect of EGCG on hepatocytes, a study revealed that EGCG suppressed fibrosis, oxidative stress, and inflammation via PI3K/Akt and an array of signaling molecules in a nonalcohol fatty liver rat model [26]. To date, the role of EGCG in protecting bovine hepatocyte inflammatory and oxidative stress induced by LPS remains unclear. Pretreatment with EGCG abolished the activity of NF-κB cascades and IFN-γ in both the plasma and liver of concanavalin-A-induced hepatitis in mice [27]. Furthermore, EGCG acts as an antioxidant through scavenging ROS, attenuating the effect of ROS accumulation by stimuli induction [28,29]. However, whether EGCG treatment is responsible for the improvement of antioxidative enzymes in bovine hepatocytes and must be further assessed. The current study investigated the role of EGCG in attenuating the LPS-induced detrimental effect on bovine hepatocytes with regard to the suppression of inflammatory responses and oxidative stress as well as restored liver damage in mice. The results may provide insights into the development of strategies against endogenous or exogenous LPS-induced hepatic stress in dairy cows as well as endotoxin-related liver diseases.

## 2. Materials and Methods

### 2.1. Chemicals

(-)-Epigallocatechin-3-gallate was commercially purchased from Aladdin (E107404, Aladdin, Shanghai, China), with a purity of more than 98%. The lipopolysaccharide used in this study was derived from *Escherichia coli* 026:B6 lyophilized powder (L6529, Sigma, St. Louis, MO, USA) and GalN was purchased commercially with a purity of more than 99% (G0500, Sigma, St. Louis, MO, USA).

### 2.2. Ethics

The experimental procedures were approved by the Animal Experiment Committee of Yangzhou University according to the Regulations for the Administration of Affairs Concerning Experimental Animals in China. The experimental procedures were strictly implemented according to the approved guidelines and regulations.

### 2.3. Cell Culture Conditions

The primary bovine hepatocytes were obtained as a gift from Prof. Juan J. Loor (Department of Animal Sciences and Division of Nutritional Sciences, University of Illinois at Urbana-Champaign) and cultivated as previously described [30]. Briefly, cells were isolated from bovine liver tissue at mid-lactation period of Holstein cows. All experiments were performed at passages of four to six generations. Hepatocytes were cultured on a 6-well plate with complete medium containing 90% RPMI 1640 Basic (8119417 Gibco, Carlsbad, CA, USA) and 10% fetal bovine serum (FBS). Antibiotics were supplemented in the medium to prevent environmental pathogen contamination (penicillin 100 IU/mL; streptomycin 100 μg/mL). Cells were cultured and maintained at 37 °C in a fixed humidified 5% CO_2_ incubator.

### 2.4. In Vitro Experimental Design

In the dose-dependent experiment for EGCG, cells were treated with EGCG at concentrations of 0, 10, 20, 50, 100, and 200 μM for 24 h. Meanwhile, the EGCG treatments of hepatocytes were conducted for 0, 6, 12, 24, and 48 h at a concentration of 50 μM. The optimized concentration and timing of EGCG treatment to cells were selected as 24 h at a concentration of 50 μM. The LPS treatment was optimized and performed as previous described, with some modifications [30]. Cells were pre-cultured with serum-fasting medium for 12 h and the formal treatments were as follows: complete medium (RPMI 1640 with heat-inactivated FBS) pretreatment for 30 h as control group (NC), complete medium pretreatment for 24 h followed by 6 μg/mL of LPS challenge (LPS) for 6 h, and pretreatment with 10 or 50 μM of EGCG for 24 h followed by 6 μg/mL of LPS challenge (LPS) for 6 h (E(10) + LPS; E(50) + LPS).

### 2.5. Cell Viability

CCK-8 assay was conducted for the determination of cell viability under different doses of EGCG using a commercial CCK-8 Cell Counting kit (Vazyme, Nanjing, China). The experiment was performed in accordance with the manufacturer’s protocols. In brief, cells (1 × 10^5^ cells/mL) were seeded onto a 96-well microplate for incubation at 37 °C for 24 h. Then, hepatocytes with specific treatments in each well were incubated with 10 mL of CCK-8 at 37 °C for 2 h. The fluorescence intensity was recorded at OD_450 nm_ using a microplate reader (SPARK, TECAN, Männedorf, Switzerland).

### 2.6. Apoptotic Rate Measurement by Flow Cytometry

Cells were seeded onto 6-well plates (2 × 10^5^ cells/mL) and treated with the selected approaches in this study. The apoptotic effect of EGCG and LPS on hepatocytes differentiation was evaluated by using Annexin V-FITC/PI Apoptosis Detection Kit (Vazyme, Nanjing, China). The staining and preparation of the cells were performed according to the instructions. The flow cytometry was obtained and analyzed using the FACSCalibur platform (BD Biosciences, Franklin Lakes, NJ, USA).

### 2.7. 5-Ethynyl-2′-Deoxyuridine (EdU) Determination

As described previously [19], the BeyoClick EdU cell proliferation kit with Alexa Fluor 555 (Beyotime, Shanghai, China) was applied for the measurement of hepatocytes proliferation under different treatments. First, 10 μM EdU solution was used to incubate cells for 2 h. Then, 4% paraformaldehyde and 0.3% Triton X-100 were used for fixation and permeabilization of the cells, respectively. Cells nuclei were stained with Hoechst for 10 min at RT and free of light. Proliferation was recorded by imaging with a fluorescence microscope DMi8 Microsystems GmbH (Leica, Wetzlar, Germany).

### 2.8. Cell Migration Assay

Migration of bovine hepatocytes was evaluated by a scratch-wound assay in which 6-well confluent cells were linearly wounded by scraping with micropipette tip after selected treatments. The gap area of the cells was observed at 0, 12, and 24 h after wounding. Images were captured using a phase-contrast microscope (Nikon, Tokyo, Japan). The rate of the wound closure was measured using Image J software (LOCI). Triplicates were done in each individual experiment.

### 2.9. ROS, MDA, GSH-Px, and T-AOC Detection

The production of ROS in all groups of cells was determined using intracellular staining with 2, 7-dichlorofuorescin diacetate (DCFH-DA) kit (Beyotime, Shanghai, China) and captured by DMi8 Microsystems BmbH (Leica, Wetzlar, Germany) and microplate reader (SPARK, TECAN, Switzerland), respectively. MDA, GSH-Px, and T-AOC were determined by using spectrophotometric diagnostic kit according to manufacturer’s instructions (Jiancheng Tech., Nanjing, China), Absorbance was recorded at 532 nm (MDA), 420 nm (GSH-Px), and 520 nm (T-AOC), respectively.

### 2.10. RNA Isolation and Quantitative Real-Time PCR

Total RNA from cells was extracted with RNA-easy Isolation Reagent (Vazyme, Nanjing, China) according to the manufacture’s instruction. The quality of the exacted RNA was evaluated using a 2100 bioanalyzer (Agilent Technologies, Santa Clara, CA, USA) and the RQN was >7.0 for all samples. cDNA was generalized using HiScript III RT SuperMix (R323-01, Vazyme, China) and qRT-PCR was performed using AceQuniversal SYBR Master Mix for qPCR (Vazyme, China) on an ABI QuantStudio PCR system (Applied Biosystems, Foster City, CA, USA). Primers used in this study was illustrated in earlier publications [17,31]. GAPDH, UXT, and RPS9 were selected as internal control genes. The target genes expression data were normalized using the geometric mean of internal control genes. The availability of the internal genes was tested in hepatocytes by previous reports [32]. The 2^~ΔΔCt^ approach was utilized for relative quantification [33].

### 2.11. Immunoblotting

Immunoblotting was essentially conducted as previously described [30]. In brief, total protein was extracted from bovine hepatocytes using RIPA lysis buffer (Beyotime, Shanghai, China). The concentration of the proteins extracted was quantified with BCA methods (Pierce, Rockford, IL, USA). Equal amounts of protein were adjusted for separation on 10% SDS polyacrylamide gels. The separated proteins were transferred onto nitrocellulose membranes (Millipore, Billerica, MA, USA) and incubated with primary antibodies (Cell signaling technology) overnight at 4 °C on the rotator. The primary antibodies for *p*-*p*65, *p*65, *p*-IκB, IκB, IL-1β, *p*-JNK, JNK, *p*-ERK, ERK, *p*-*p*38, *p*38, *p*-c-Jun, c-Jun, ICAM-1, MCP-1, Nrf2, NQO1, HMOX1, and GAPDH were purchased from Cell Signaling Technology (#3033, #8242, #2859, #4812, #4668, #9252, #4370, #4695, #4511, #8690, #3270, #9165, #67,836, #81,559, #12,721, #62,262, #26,416, and #5174) and diluted as 1:1000. After moderate washing with TBST, the blots were incubated with horseradish peroxidase-coupled secondary antibodies (#7074, CST) and diluted as 1:5000. The intensity of each blot was normalized with quantification of GAPDH. The blots were analyzed and quantified with Image J software (LOCI).

### 2.12. Chromatin Immunoprecipitation

The experiment was performed according to the protocols described previously [34]. Briefly, cells selected after treatment were harvested using PBS containing protease inhibitor cocktail (Cat. 11697498001; Roche, Basel, Switzerland). Protein and DNA were cross-linked by adding 1% formaldehyde. The reaction was terminated by glycine after shaking for 10 min. The mixture was then centrifuged at 4 °C, 4000× *g* for 5 min. The chromatin was then sonicated into 200 to 500 bp on ice. Then, 4 μg of primary antibody (Anti-*p*65, ab16,502, Abcam, Cambridge, UK) was utilized for incubation with chromatin preparations overnight at 4 °C. IgG raised from rabbit was used as a negative control. Protein A/G agarose beads (40 μL, 50% slurry, sc-2003; Santa Cruz Biotechnology, Dallas, TX, USA) was used to acquire immunoprecipitation of chromatin complexes and took 200 μL of the sample as input. Promoter fragments obtained from chromatin immunoprecipitation were then quantified using a qPCR system with specific primers for *TNFA* and *IL1B* promoter motif (*TNFA*: forward 5′GACAGAAGGTG TAGGGCCAG 3′ and reverse 5′CGCTCTGGGAGCTTCTCT 3′; *IL1B*: forward 5′GGCT CAGCTTGTAAAGAATC 3′ and reverse 5′GAATGCACGAAAGTC ATCC 3′).

### 2.13. Immunofluorescence

Cells were seeded onto a 12-well plate attached with crawling slides (φ 20 mm). After the treatment, 4% paraformaldehyde was used to fix cells for 15 min, and washed with PBS three times, and incubated with 0.5% Triton-X for 15 min at RT. Slides were then incubated with 5% BSA at 37 °C for 1 h blocking. Primary antibodies (targeting the proteins of interest) were used to incubate with cells containing 1% BSA and 0.3% Triton X-100 (T9284, Sigma-Aldrich, St. Louis, MO, USA) at 4 °C overnight. FITC-labeled secondary antibody was used to stain the cells. After being washed three times with PBS, DAPI (1 μg/mL, D8417, Sigma-Aldrich, St. Louis, MO, USA) was used to detect nuclei for 5 min at RT. The images were captured using DMi8 Microsystems BmbH (Leica, Wetzlar, Germany).

### 2.14. Animals

Six to eight-week-old BALB/c mice were obtained from the Center of Comparative Medicine in Yangzhou University. The mice were allowed to adapt to the standardized environmental conditions (23 ± 2 °C; 55 ± 10% of humidity) for one week prior to infection. Animal experiments were performed in accordance with approved guidelines and regulations (YZU-2021003-164). The animal usage license was certified by Jiangsu Science and Technology Department and the number is SYXK-2017-0044.

### 2.15. In Vivo Experimental Design

Mice (50 in total) were divided into following groups each comprising of six mice:

1. **Control group**: mice were treated normally as control;
2. **GalN/LPS group**: mice received intraperitoneal injection with GalN (700 mg/kg) and LPS (10 μg/kg) at day 11 for 6 h;
3. **EGCG + GalN/LPS group**: mice were administrated with EGCG orally at a dose of 10, 25, or 50 mg/kg/day for 10 days, followed by GalN/LPS challenge at day 11 for 6 h.

Animals were sacrificed under ether anesthesia at 6 h post-GalN/LPS challenge by cervical dislocation. The eyeballs were removed for collecting the peripheral blood in anticoagulation tubes (EDTA). Livers were excised and rinsed in cold PBS, and all the samples were quickly stored at −80 °C.

### 2.16. Lethality Determination

The survival rates were monitored for a period of 24 h in groups as selected in this study. The time points for counting the dead mice were at 8 h, 12 h, 18 h, and 24 h after GalN/LPS injection.

### 2.17. Histology Determination

Liver tissues obtained aseptically from all experimental groups were cut into pieces and fixed in 10% buffered formalin phosphate. Then, samples were paraffin-embedded, sectioned, and stained with hematoxylin–eosin and examined using a phase-contrast microscope (Nikon, Tokyo, Japan).

### 2.18. Aminotransferase Activities, Lipid Peroxidation, and GSH-Px Content

The activities of alanine aminotransferase (ALT) and aspartate aminotransferase (AST) were examined at 6h after GalN/LPS stimulation according to the ALT and AST ELISA kit instructions (ab282882 and ab263882, Abcam, Cambridge, UK). The concentrations of MDA and GSH-Px in the mouse plasma from all the groups were determined by ELISA analysis. The experiment was conducted using a commercially available kit following the manufacturer’s instructions (Nanjing Jiancheng Bioengineering Institute, Nanjing, China).

### 2.19. Statistical Analysis

In the dose—and timingresponse experiments, the linear, quadratic effects of EGCG on all response criteria were evaluated using contrast analysis. Abundance data (mRNA and protein) were log^−2^ transformed prior to analysis to fit normal distribution of residuals. The resulting LS means were log^−2^ back-transformed for ease of interpretation and reported in figures. Data were analyzed using one-way ANOVA with Dunnett’s post-test by SAS Statistics (v. 9.2, SAS Institute Inc., Cary, NC, USA). Data were expressed as means ± S.E.M. Differences with *p* values < 0.05 were considered as statistically significant. Experiments were performed in triplicate.

## 3. Results

### 3.1. The Optimized EGCG Dose Was Determined by Cell Viability and TNF-α Concentration

**Figure 1:**
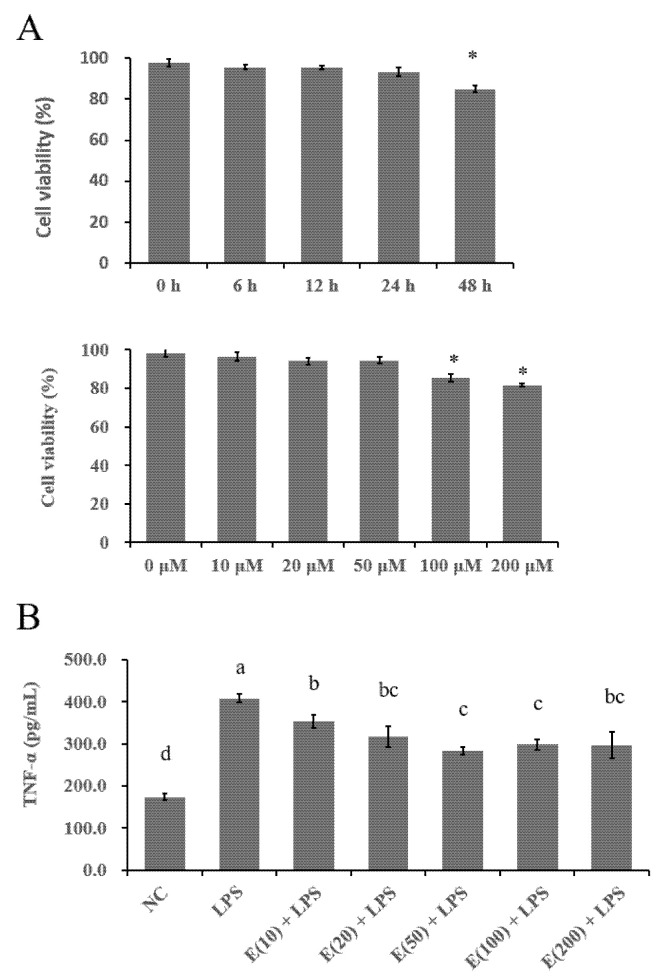
Cell viability and TNF-α secretion of cells treated with different concentrations of EGCG followed by LPS-challenge. Cells were treated with EGCG at concentrations of 0, 10, 20, 50, 100, and 200 μM for 24 h. Meanwhile, the EGCG treatments of hepatocytes were conducted for 0, 6, 12, 24, 48 h at a concentration of 50 μM. Absorbance for cell viability or TNF-α was measured at 450 nm. The results are expressed as means ± S.E.M. * *p* < 0.05 vs. the control group (**A**), and the letters in superscript indicate that the difference between groups was significant (*p* < 0.05) (**B**). Data are representative of three independent replicates.

Cell viability was affected by different doses and timings of EGCG treatment and was determined using a CCK-8 assay. As shown in Figure 1A, for the dose experiment, both 100 and 200 μM EGCG reduced cell viability compared to the control (0 μM) group (*p* < 0.05). Regarding the timing of the EGCG treatment on hepatocytes, 50 μM EGCG did not affect cell viability within 24 h, whereas 48 h of EGCG treatment decreased the rate of viable cells (*p* < 0.05). The concentration of TNF-α in the cell-culture medium was elevated by LPS challenge at a dose of 6 μg/mL. However, pretreatment with EGCG at doses ranging from 10 to 200 μM decreased the production of TNF-α induced by LPS stimulation compared with that in the LPS group (*p* < 0.05) (Figure 1B). Among the effective doses and timing of EGCG evaluated in bovine hepatocytes, pretreatment of the cells with 50 μM EGCG for 24 h was selected as the optimized concentration in this study.

### 3.2. Impaired Cell Proliferation Was Restored by Pretreatment with EGCG

**Figure 2:**
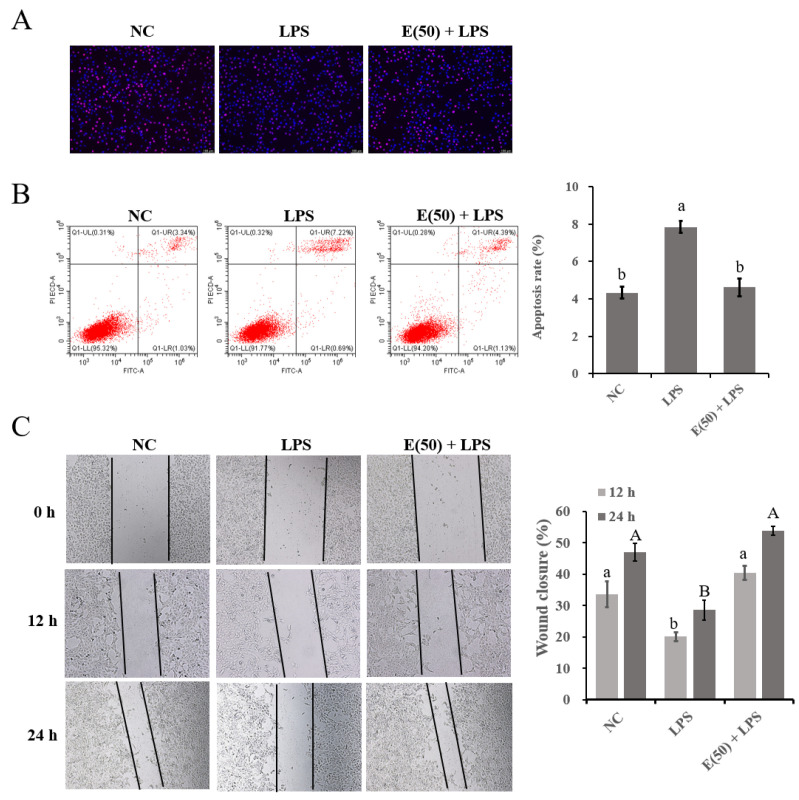
The effect of EGCG pretreatment on cell proliferation followed by LPS stimulation. The concentration and timing of EGCG treatment of cells were selected as 24 h at a concentration of 50 μM. (**A**) EdU determination for cell proliferation. Cells were observed at 200× magnification. (**B**) Apoptotic cells were examined using flow cytometry. Apoptotic rate were calculated with the value of early—and late—apoptotic cells. (**C**) Wound-healing assay was performed for the migration ability of hepatocytes and the closure rate was imaged at 0, 12, and 24 h after scraping. Cells were pretreated with EGCG at a dose of 50 μM for 24 h, followed by the induction of 6 μg/mL LPS for 6 h. Results are expressed as means ± S.E.M. Letters in lowercase indicate differences between groups at 12 h, while uppercase indicates differences between groups at 24 h (*p* < 0.05).

The proliferation of cells exposed to different treatments in this study was determined by EdU staining, flow cytometry, and scratch-wound assays (Figure 2). LPS challenge weakened the EdU staining in the LPS group, whereas cells pretreated with EGCG at 50 μL following LPS stimulation restored the rate of hepatocyte proliferation as demonstrated by increased staining (Figure 2A). The number of apoptotic cells in the LPS group significantly increased approximately two-fold compared to the control group (*p* < 0.05). Furthermore, pretreatment with EGCG decreased cell apoptosis in the E(50) + LPS group to a level similar to that noted in the control group (Figure 2B). Cell growth was affected by the LPS challenge as demonstrated by the reduced migration rate of bovine hepatocytes at both 12 h and 24 h in the LPS group compared to the control group (*p* < 0.05). However, cells pretreated with EGCG following LPS challenge exhibited wound closure activity similar to that noted in the control group at 12 h and 24 h (*p* < 0.05).

### 3.3. LPS-Induced Activation of the NF-κB Signaling Pathway Was Reversed by EGCG Pretreatment

**Figure 3:**
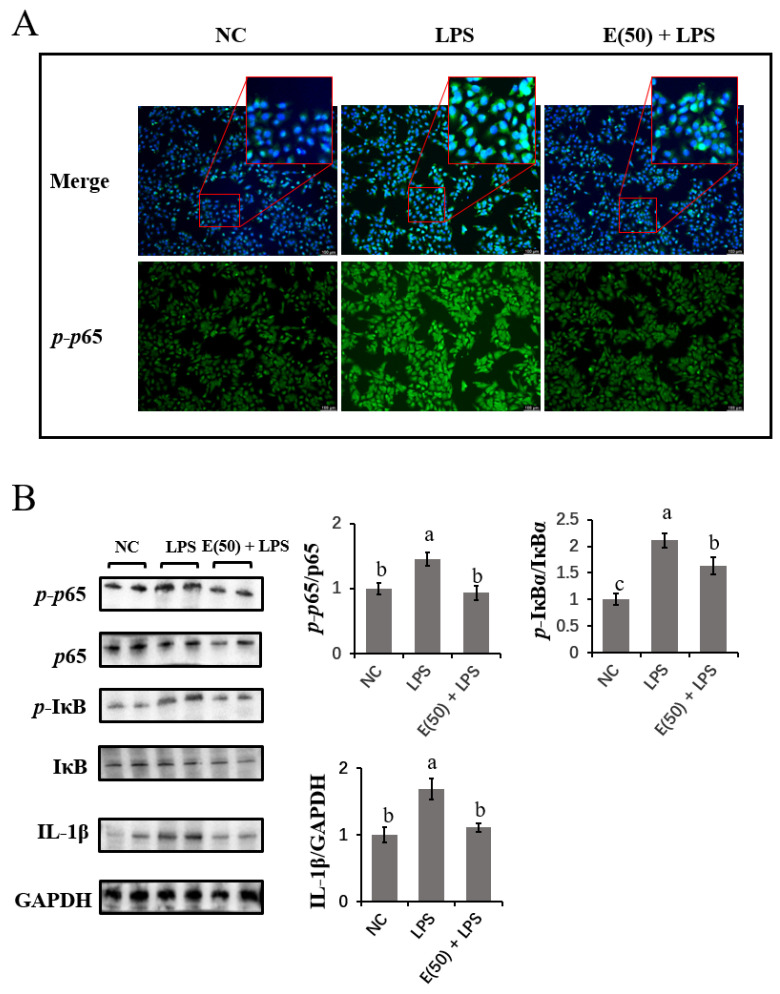
The effect of EGCG pretreatment on LPS-induced NF-κB signaling components. The concentration and timing of EGCG treatment of cells were selected as 24 h at a concentration of 50 μM. (**A**) Immunofluorescence of *p*-*p*65 proteins expressed in hepatocytes and imaged using DMi8 Microsystems BmbH. Cells were observed at 200× magnification. (**B**) Immunoblots were determined for the expression of proteins related to NF-κB signaling and pro-inflammatory cytokine. Experiments were performed as three independent replicates. Results are expressed as means ± S.E.M. Letters in superscript indicate that the difference between groups was significant (*p* < 0.05).

The translocation of the phosphorylated NF-κB subunit p65 into the nucleus was imaged by fluorescent staining with an FITC-labeled antibody. As shown in Figure 3A, the activation of *p*65 in LPS-challenged cells was reduced by EGCG pretreatment, resulting in reduced staining of *p*-*p*65 in hepatocyte nuclei. The expression of proteins related to NF-κB signaling was examined using immunoblotting (Figure 3B). The ratio of phosphorylated *p*65 to total *p*65 was upregulated by LPS stimulation compared to that in the control group (*p* < 0.05). EGCG pretreatment reversed the activation of NF-κB *p*65 by LPS as demonstrated by a reduced ratio of phosphorylated *p*65 to total *p*65 (*p* < 0.05). In addition to the suppression of NF-κB *p*65, the upregulated ratio of phosphorylated IκBα to total IκBα induced by LPS was decreased by pretreatment with EGCG following LPS challenge (*p* < 0.05). As a target cytokine that is regulated by NF-κB, IL-1β protein expression was upregulated in the LPS groups compared to the control group. However, compared to the LPS group, the upregulation of IL-1β induced by LPS challenge was blocked in cells pretreated with EGCG (*p* < 0.05).

### 3.4. EGCG Suppressed the Expression of Genes Related to Proinflammation by Reducing the Binding Activity of NF-κB

**Figure 4:**
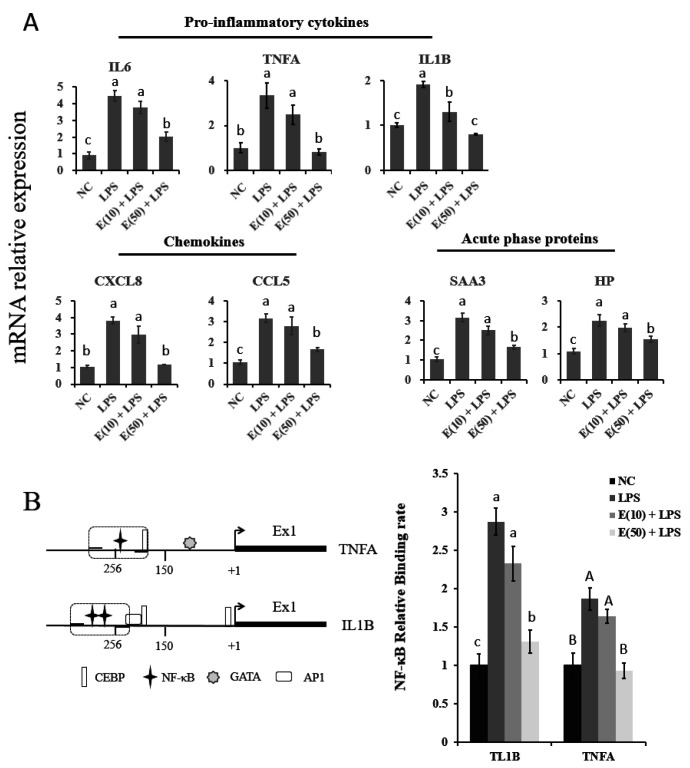
The expression of pro-inflammatory genes and binding activity of NF-κB subunit *p*65. The concentration and timing of EGCG treatment to cells were selected as 24 h at a concentration of 10 μM or 50 μM. (**A**) The mRNA expression of pro-inflammatory cytokines, chemokines, and acute-phase-protein-encoding genes. Abundance of each gene was normalized by the geometric mean of the internal control genes (*GAPDH*, *UXT,* and *RPS9*). Abundance of genes in the NC group was set as 1.0. (**B**) Numbers refer to the position relative to the transcriptional starting site, indicated by black arrows. The position of the transcription factors is indicated by the respective symbols. The positions of primers used for chromatin immunoprecipitation assays are denoted by dashed lines surrounding the symbol of NF-κB. The identification of target promoter regions of candidate genes was determined by BLAST analysis as DNA-sequences that are 5′-upstream of the mRNA sequences deposited in the NCBI: NM_174093.1 (*IL1B*); NM_173966.3 (*TNFA*). Level of NF-κB binding to *IL1B* and *TNFA* promoter. Letters in lowercase indicate differences between groups for *IL1B*, while uppercase indicate differences between groups for *TNFA* (*p* < 0.05). Results are expressed as means ± S.E.M.

The expression of genes related to proinflammatory cytokines, chemokines, and acute-phase proteins was examined using quantitative PCR, as shown in Figure 4A. Cells challenged with LPS showed significantly upregulated expression of *IL6*, *TNFA*, *IL1B*, *CXCL8*, *CCL5*, *SAA3*, and *HP* compared to those in the control group (*p* < 0.05). *IL6*, *CXCL8*, *CCL5*, *SAA3*, and *HP* mRNA levels in cells pretreated with 10 μM EGCG prior to LPS stimulation did not differ from those observed in the LPS group. However, compared to the LPS group, pretreatment with 50 μM EGCG markedly reduced the upregulation of all these genes to levels similar to those of the control group.

The binding activity of NF-κB p65 to the *IL1B* and TNFA promoters was determined using a ChIP–qPCR assay, as shown in Figure 4B. The putative binding position for NF-κB on the *IL1B* and *TNFA* promoters was analyzed using PROMO software (http://alggen.lsi.upc.es/cgi-bin/promo_v3/promo/promoinit.cgi?dirDB=TF_8.3, accessed on 5 March 2018) with a threshold of filters > 0.90. LPS challenge induced the binding of NF-κB to both the *IL1B* and *TNFA* promoter motif sequences compared to that in the control group (*p* < 0.05). In contrast, pretreatment with 50 μM EGCG but not 10 μM blocked the LPS-induced activation of NF-κB activity, which is consistent with the increased translocation of NF-κB *p*65 into the nucleus.

### 3.5. The Inhibitory Effect of EGCG on MAPK Signaling Pathway Activity in Hepatocytes

**Figure 5:**
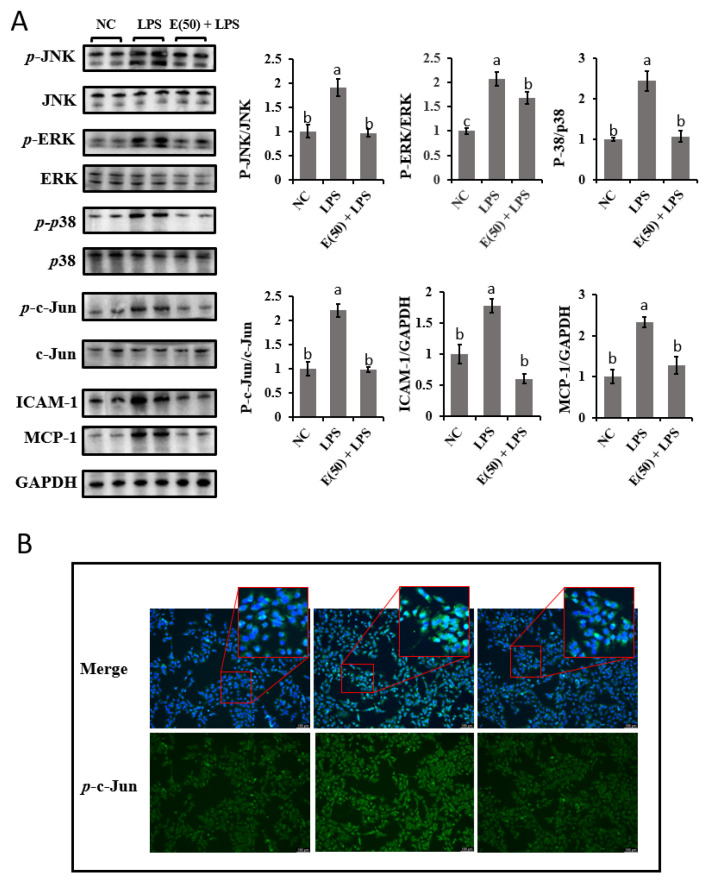
The effect of EGCG pretreatment on LPS-induced activities of MAPK-signaling-involved components. The concentration and timing of EGCG treatment to cells were selected as 24 h at a concentration of 50 μM. (**A**) Immunoblots and calculated gray intensity for the evaluation of phosphorylated level of MAPK signaling proteins. For ICAM-1 and MCP-1, protein expression was normalized by comparing with GAPDH. (**B**) Immunofluorescence was determined for the import of phosphorylated c-Jun into the nucleus. Cells were observed at 200× magnification. Results are expressed as means ± S.E.M. Letters in superscript indicate that the difference between groups was significant (*p* < 0.05). Experiments were performed as three independent replicates.

To evaluate the responses of MAPK signaling to EGCG and LPS treatment in bovine hepatocytes, the protein expression of the MAPK pathway signaling components JNK, ERK, and *p*38 and the downstream effectors c-Jun, ICAM-1, and MCP-1 were examined (Figure 5A). LPS challenge activated MAPK signaling as a result of the upregulated abundance of phosphorylated JNK, ERK, and *p*38 compared to the control group (*p* < 0.05). In contrast, pretreatment with EGCG decreased the ratio of phosphorylated JNK, ERK, and *p*38 to total JNK, ERK, and *p*38, respectively, compared to the LPS group (*p* < 0.05). Moreover, the increased ratio of phosphorylated c-Jun to total c-Jun was decreased by pretreatment with EGCG following LPS challenge compared to the LPS group. This finding is consistent with the results of phosphorylated JNK protein expression. As downstream targets of c-Jun, chemokines, including ICAM-1 and MCP-1, were upregulated by LPS stimulation, whereas pretreatment with EGCG reversed the production of these proteins in bovine hepatocytes (*p* < 0.05). The translocation of phosphorylated c-Jun into the cell nucleus was observed by immunofluorescence (Figure 5B). Compared with control cells, the higher predominant nuclear localization of c-Jun confirmed the activation of c-Jun in the LPS group. However, significant inactivation of c-Jun was observed in the cells pretreated with EGCG following LPS challenge.

### 3.6. The Oxidative Stress Induced by LPS Was Reversed by Supplementation with EGCG in Hepatocytes

**Figure 6:**
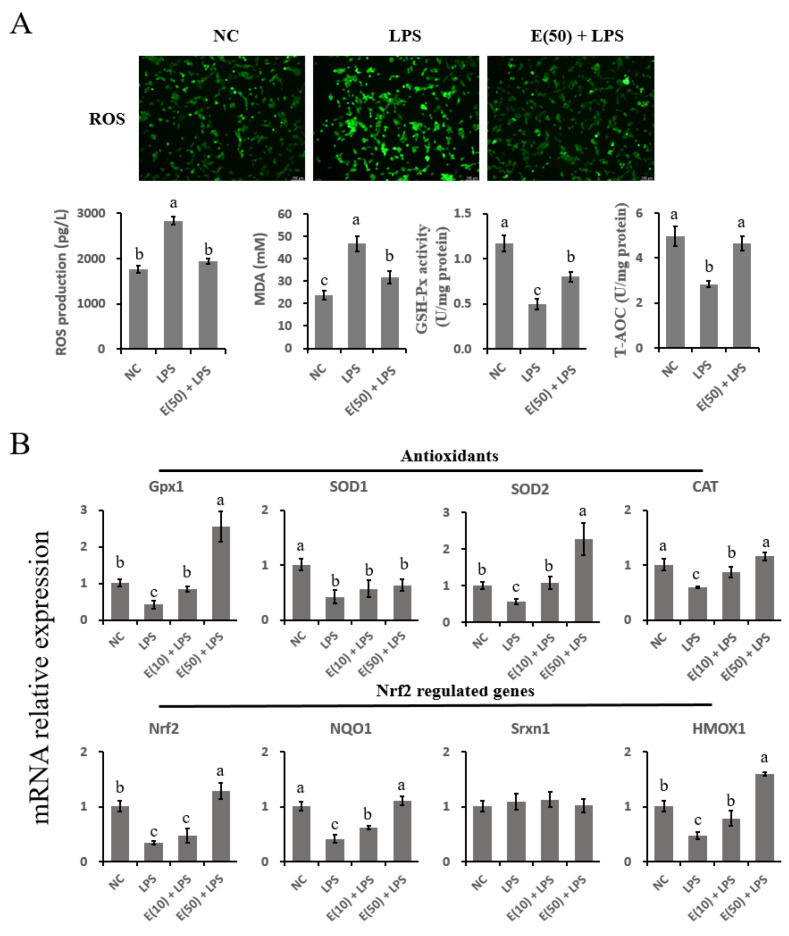
Antioxidant capacity was affected by the EGCG pretreatment and LPS stimulation in hepatocytes. The concentration and timing of EGCG treatment to cells were selected as 24 h at a concentration of 10 μM or 50 μM. (**A**) DCFH-DA probe for intracellular ROS content was captured and determined. Cells were observed at 200× magnification. The absorbance of intensities for ROS, MDA, GSH-Px, and T-AOC were obtained at 525 nm, 532 nm, 420 nm, and 520 nm. (**B**) The expression of genes related to antioxidants and Nrf2 signaling. Abundance of each gene was normalized by the geometric mean of the internal control genes (*GAPDH*, *UXT*, and *RPS9*). Abundance of genes in the NC group was set as 1.0. Results are expressed as means ± S.E.M. Experiments were performed as three independent replicates. Letters in superscript indicate that the difference between groups was significant (*p* < 0.05).

To confirm that oxidative stress was induced by LPS, the production of ROS and the antioxidant capacity of the cells were studied, as shown in Figure 6A. LPS treatment enhanced ROS production as demonstrated by increased staining of intracellular ROS and concentrations of ROS or MDA in the LPS group. However, pretreatment with 50 μM EGCG inhibited the accumulation of ROS and MDA induced by LPS challenge. Regarding antioxidant enzyme activity, GSH-Px and T-AOC activity were reduced by LPS stimulation, whereas EGCG pretreatment improved the antioxidant capacity by increasing the GSH-Px activity and T-AOC compared to the LPS group (*p* < 0.05).

The expression of genes related to antioxidant and Nrf2 signaling was determined using quantitative PCR (Figure 6B). LPS challenge impaired the antioxidant capacity of hepatocytes by reducing the expression of antioxidant enzyme-encoding genes (*Gpx1*, *SOD1*, *SOD2*, and *CAT*) (*p* < 0.05). Pretreatment with 50 μM EGCG restored the suppression of *Gpx1*, *SOD2*, and *CAT* gene expression (*p* < 0.05). However, the expression of the *SOD1* gene in both the E(10) + LPS and E(50) + LPS groups was not affected compared to that in the LPS group (*p* < 0.05). For genes related to Nrf2 signaling, LPS stimulation downregulated *Nrf2*, *NQO1*, and *HMOX1* expression compared to the control group (*p* < 0.05). In contrast, the abundance of the *Nrf2*, *NQO1*, and *HMOX1* genes in cells pretreated with EGCG at both 10 and 50 μM was greater than that in the LPS group (*p* < 0.05). *Srnx1* mRNA expression was not altered by LPS challenge or by EGCG pretreatment (*p* > 0.05).

### 3.7. EGCG Enhanced the Antioxidant Capacity of Hepatocytes by Activating Nrf2 Signaling

**Figure 7:**
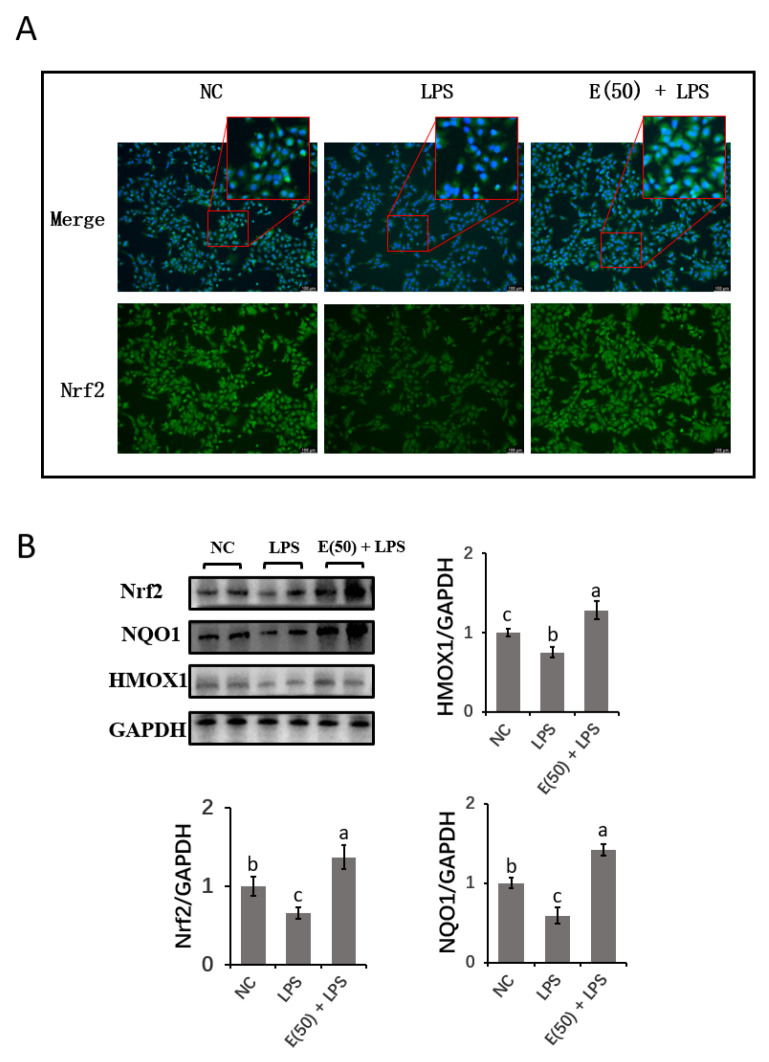
Protein expression of Nrf2 signaling components. The concentration and timing of EGCG treatment to cells were selected as for 24 h at a concentration of 50 μM. (**A**) Immunofluorescence for detecting the Nrf2 molecules in cells nuclear. The target protein was stained with FICT-labeled secondary antibody (Green), and the DAPI was used for staining nuclear (Blue). Cells were observed at 200× magnification. (**B**) Nrf2 signaling related proteins were determined using western blot immunoassay. Abundance of each protein was normalized with GAPDH band intensities in corresponding group. Results are expressed as means ± S.E.M. Experiments were performed as three independent replicates. Letters in superscript indicate that the difference between groups was significant (*p* < 0.05).

We further studied the expression of proteins related to Nrf2 signaling (Figure 7). The results showed that LPS inhibited the nuclear staining of Nrf2, and inhibition of Nrf2 translocation into the nucleus was reversed in EGCG-pretreated cells as demonstrated by increased staining. Nrf2, NQO1, and HMOX1 protein expression followed a similar pattern as that noted for gene expression. A significant decrease in Nrf2, NQO1, and HMOX1 protein expression was also observed in the LPS group compared to the control group (*p* < 0.05). In contrast, EGCG treatment counteracted the LPS-induced suppression of Nrf2, NQO1 and HMOX1 protein expression (*p* < 0.05).

### 3.8. Lethality in Mice and Histological Changes in Liver Tissue

**Figure 8:**
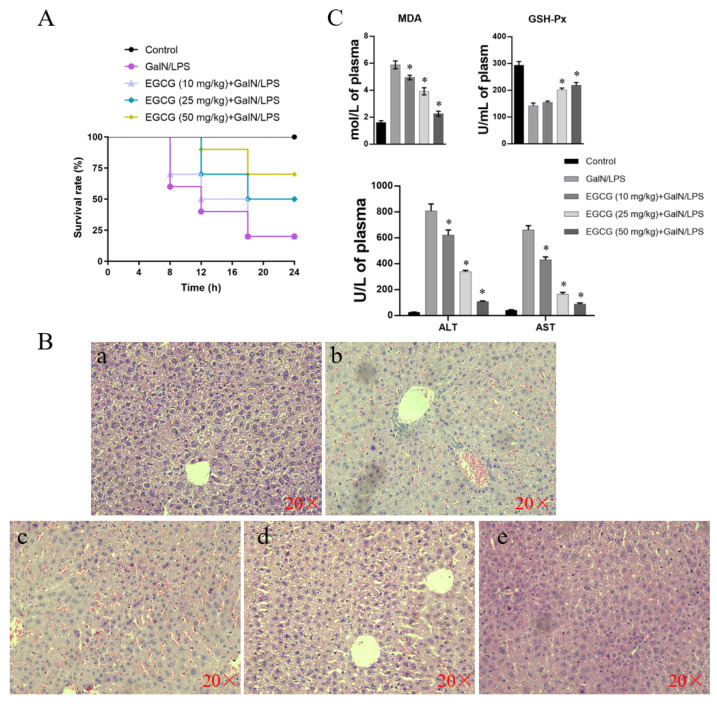
The effect of EGCG on the GalN/LPS-induced mortality and liver damage in mice. (**A**) Mortality of mice administrated with EGCG followed by GalN/LPS challenge. Mice were intraperitoneally injected with GalN/LPS, with oral administration of EGCG at (10, 25, and 50 mg/kg) for 10 days before GalN/LPS injection. (**B**) H&E staining of the liver after GalN/LPS injection. Images from each experimental group were original magnification of ×400. (**a**) Control group; (**b**) mice treated with GalN/LPS; (**c**–**e**) EGCG treated (10, 25, and 50 mg/kg) followed by GalN/LPS injection. (**C**) Plasma levels of MDA, GSH-PX, and ALT and AST in all groups of mice. * *p* < 0.05 vs. the GalN/LPS group. Results are expressed as means ± S.E.M.

As shown in Figure 8A, the mice received standard feed 6 h after GalN/LPS injection. The mortality was 80% at 24 h, but this rate decreased upon pretreatment with EGCG at 25 and 50 mg/kg. Mice pretreated with 50 mg/kg ECGC exhibited 20% viability, representing a four-fold reduction compared to the GalN/LPS group. The survival curves were significantly different using the log-rank test (*p* < 0.01). Regarding histological changes in the mouse liver, the captured images in Figure 8B show normal structure and lobular architecture in the liver of control animals. However, mice exposed to GalN/LPS exhibited significant hemorrhagic necrosis, portal inflammation, hyperemia, and inflammatory cell infiltration. Liver damage and disorder was prevented by pretreatment with 50 mg/mL EGCG.

### 3.9. Analysis of Peripheral Aminotransferase Activity and GSH-Px and MDA Contents

As shown in Figure 8C, serum ALT and AST levels in the vehicle group were lower than those in the mice that received injection of GalN/LPS after 6 h. Reduction in ALT and AST were observed when EGCG was administered to mice at concentrations from 10 to 50 mg/kg. Similarly, the increased MDA content in the GalN/LPS group was decreased by administering mice with increasing amounts of EGCG. Moreover, levels of the antioxidant enzyme GSH-Px were reduced by GalN/LPS treatment in the mice, whereas the antioxidant capacity was restored by the administration of 50 mg/kg EGCG.

## 4. Discussion

LPS-induced inflammatory and oxidative responses have been reported in many cell types and animal models. In bovine liver, increased circulating endogenous or exogenous LPS impairs the hepatic immunity and capacity for free radical elimination. In this study, the protective effect of EGCG on LPS-challenged hepatocyte inflammation and oxidative responses occurred by targeting specific molecules through the inhibition of NF-κB and MAPK signaling and improvements in Nrf2 signaling were demonstrated. EGCG neutralized the detrimental effect of LPS on the dysregulation of cell proliferation. In addition, the administration of EGCG in mice was demonstrated to be beneficial to combating the hepatic proinflammatory responses or oxidative stress induced by stimuli, such as GalN/LPS.

### 4.1. LPS-Impeded Proliferation Is Restored by EGCG Pretreatment

As one of the main functions in bovine liver, an excessive accumulation of endotoxins beyond the clearance ability has been reported to induce the liver injury [17]. The excess exposure to these negative stimuli may lead to hepatic cell apoptosis and impede the detoxification capacity of antioxidants [35,36]. It has been shown that increased concentration of LPS and its prolonged incubation time, or the interaction between the two, results in the trend towards a decrease in bovine mammary epithelial cell proliferation [37]. Moreover, LPS drive the response of increased liver injury, hepatocyte apoptosis, and macrophage infiltration, as well as decreased cell proliferation in a *Polygonum multiflorum*-induced hepatotoxic rat model [38]. Notably, several studies found that EGCG restrained the proliferation and migration of cancer cells or hepatic stellate cells through inhibiting angiogenesis or collagen synthesis. However, in the current study, the apoptotic cells and decrease of cell proliferation induced by LPS challenge were restored by the pretreatment with EGCG. Moreover, the promotive effect of EGCG on bovine hepatocytes proliferation is in agreement with the study that tea polyphenols enhanced the survival of bovine mammary cells [13]. This may support that EGCG affect cells with a high proliferative and metabolic rate in a cell-type-specific fashion.

### 4.2. EGCG Mediated Inactivation of the NF-κB Signaling Pathway in Hepatocytes

To detect the signs of proinflammatory molecule alterations, the components of the NF-κB signaling pathway, a well-known signaling pathway involved in LPS-induced inflammatory responses, was monitored. As IκBα is phosphorylated in the cytoplasm, the silent complex of NF-κB primarily departs from IκBα and primarily translocates into the nucleus [39]. These effects subsequently induce a master switch to the regulation of proinflammatory cytokines, such as IL1B, TNFA, and IL6 [40]. Circulating LPS from either endogenous conditions, such as ruminal acidosis, or experimentally infused into the udder have been proposed to contribute to the hepatic acute-phase response (APR) with signs of elevated serum amyloid A (SAA3) and haptoglobin (HP) in cattle [41,42]. EGCG is suggested to intervene against multiple pathological or physiological processes via inhibition of NF-κB signaling in the case of liver fibrosis and osteogenesis promotion [3,43,44]. In the present study, EGCG pretreatment downregulated the LPS-induced activation of NF-κB signaling components in bovine hepatocytes. This finding is consistent with the results indicating that pretreatment with EGCG reduced the translocation of phosphorylated *p*65 into the nucleus and led to the interrupted transcription of proinflammatory cytokines, chemokines, and APR genes. Furthermore, the decreased binding activity of NF-κB to the *IL1B* and *TNFA* promoters contributed to the EGCG-mediated suppression of LPS-stimulated *IL1B* and *TNFA* gene expression. The results confirmed that NF-κB signaling inactivation is involved in the effect of EGCG on immune gene expression in bovine hepatocytes.

### 4.3. EGCG Restricts LPS-Induced Inflammation by Silencing MAPK Signaling

In addition to NF-κB signaling activation, LPS induces the cascades of proinflammatory responses through the modulation of the MAPK transduction pathway in murine macrophages and bovine mammary epithelial cells [19,45]. As such, LPS stimulation in this study was observed to increase the phosphorylated levels of the MAPK signaling proteins *p*38, ERK, and JNK in bovine hepatocytes. However, EGCG pretreatment attenuated MAPK-signaling activation, as evidenced by the decreased phosphorylation of MAPK signaling proteins and the transcription factor AP-1 in response to LPS challenge. Although the interaction between MAPK and transcription factors, such as AP-1, is unknown in bovine hepatocytes, accumulating evidence indicates that LPS-induced responses of inflammation are associated with MAPK/AP-1 signaling cascades [46,47,48]. AP-1 is a dimeric complex protein and comprises Jun and Fos subfamilies. The level of c-Jun phosphorylation and translocation into the nucleus indicates activation of AP-1, which binds to a DNA consensus sequence [49,50]. The MAPK pathways regulate the phosphorylation of AP-1 proteins and subsequently control the production of inflammatory cytokines and chemokines in response to environmental stress, such as LPS challenge [51]. In the present study, EGCG pretreatment suppressed AP-1 activity by reducing MAPK signaling during LPS stimulation. Moreover, chemokines, including MCP-1 and ICAM-1, were downregulated by EGCG pretreatment followed by LPS challenge. This finding is consistent with the observation of greater import of phosphorylated c-Jun in the hepatocyte nucleus. These results indicate that EGCG suppresses inflammation through both MAPK and NF-κB signaling followed by the inhibition of inflammatory cytokine and chemokine secretion.

### 4.4. Antioxidant Capacity Was Improved by EGCG Treatment in Bovine Hepatocytes

Studies have shown that LPS induces oxidative damage via the accumulation of oxygen-free radicals, lipid peroxides, and aldehyde peroxide compounds, such as ROS and MDA, in many cell types [52]. As indicators of oxidative damage, SOD, GSH-Px, and CAT are enzymes used to assess the antioxidant capacity of the cell [53]. During LPS-induced inflammation, macrophage sensitivity to LPS-induced generation of ROS is suggested to be regulated by the MAPK and NF-κB transduction pathways [45]. LPS decreased the activities of antioxidant enzymes in bovine hepatocytes, as evidenced in the current study. This finding is consistent with the impaired hepatic antioxidant system due to digestive-tract-released LPS, as previously reported [31]. However, EGCG prevented overproduction of ROS and MDA and dysfunction of antioxidant enzymes (GSH-Px and T-AOC) as well as the encoding genes (*Gpx1*, *SOD2*, and *CAT*) in hepatocytes in our study and others [54]. This finding is supported by the result that LPS-induced downregulation of Nrf2 signaling was reversed by EGCG pretreatment as demonstrated by increased Nrf2 translocation into the nucleus and increased abundance of Nrf2 targeting genes and enzymes (NQO1 and HMOX1). Oxidative-stress-induced hepatic injury manifests as Nrf2 activation due to crosstalk with NF-κB-mediated inflammation [55]. Consistent with this study, the reduced in NF-κB signaling mediated by EGCG may be attributed to activated Nrf2 signaling and the interrupted production of ROS. However, further studies are needed to uncover the interaction of Nrf2 signaling and NF-κB or MAPK in EGCG-mediated antioxidant activity.

### 4.5. In Vivo Study Verified the Hepatoprotective Role of EGCG in Mice

In this study, the mice treated with GalN/LPS began to die at 8 h, and a 20% mortality rate was observed by 18 h. The seriously damaged histological integrity after 6 h of GalN/LPS injection was recovered by the administration of EGCG. This damage was indicated by the elevated release of plasma ALT and AST in the GalN/LPS group, which was restricted by EGCG administration in a dose-dependent manner. Hence, EGCG is suggested to protect mice from GalN/LPS-induced acute hepatitis, as evidenced by the decreased mortality and plasma ALT and AST levels. Furthermore, treatment with EGCG attenuated a decrease in GSH-Px content and lipid peroxidation (MDA) in plasma. Oxidative stress is mitigated by host endogenous antioxidant defenses [56]. Therefore, improved GSH-Px by EGCG may contribute to overwhelming MDA and result in the reduction of oxidative damage.

## 5. Conclusions

In conclusion, LPS-induced inflammatory responses are closely regulated by NF-κB and MAPK molecules in bovine hepatocytes as well as the involvement of Nrf2 signaling in oxidative stress. EGCG significantly attenuates inflammatory reactions and oxidative stress under the control of the NF-κB and MAPK cascades and the Nrf2 complex. EGCG alleviates GalN/LPS-induced inflammatory and oxidative damage in mice. These results indicate that EGCG has a potential role in the development of therapeutic agents for hepatitis in ruminants and monogastric animals.

## Data Availability

The data presented in this study are available on request from the corresponding author.

## Abbreviations

LPS	Lipopolysaccharide
EGCG	(-)-epigallocatechin-3-gallate
MAPK	Mitogen-activated protein kinase
NF-κB	Nuclear factor kappa B
APR	Acute-phase responses
Nrf2	Nuclear factor(erythroid-derived2)-like 2 protein
AST	Aspartate aminotransferase
ALT	Alanine aminotransferase
MDA	Malondialdehyde
ROS	Reactive oxygen species
EdU	5-Ethynyl-2′-deoxyuridine
GAPDH	Glyceraldehyde-3-phosphate dehydrogenase
RPS9	Ribosomal protein S9
UXT	Ubiquitously-expressed transcript
